# Racial and ethnic differences in smoking changes after chronic disease diagnosis among middle-aged and older adults in the United States

**DOI:** 10.1186/s12877-017-0438-z

**Published:** 2017-02-08

**Authors:** Ana R. Quiñones, Corey L. Nagel, Jason T. Newsom, Nathalie Huguet, Paige Sheridan, Stephen M. Thielke

**Affiliations:** 1 OHSU-PSU School of Public Health, Oregon Health & Science University, 3181 SW Sam Jackson Park Rd, Portland, OR 97239 USA; 2 Portland VA Medical Center, Oregon Health & Science University, 3181 SW Sam Jackson Park Rd, Portland, OR 97239 USA; 3 School of Nursing, Oregon Health & Science University, 3181 SW Sam Jackson Park Rd, Portland, OR 97239 USA; 4 Department of Psychology, Portland State University, P.O. Box 751, Portland, OR 97207 USA; 5 Department of Family Medicine, Oregon Health & Science University, Portland, OR USA; 6 Geriatric Research, Education, and Clinical Center, Puget Sound VA Medical Center, 1660 South Columbian Way, Seattle, WA 98108 USA; 7 Psychiatry and Behavioral Sciences, University of Washington, 1959 Pacific Avenue, Seattle, WA 98195 USA

**Keywords:** Racial/ethnic disparities, Smoking, Behavior change, Chronic disease, Disease management

## Abstract

**Background:**

Middle-aged and older Americans from underrepresented racial and ethnic backgrounds are at risk for greater chronic disease morbidity than their white counterparts. Cigarette smoking increases the severity of chronic illness, worsens physical functioning, and impairs the successful management of symptoms. As a result, it is important to understand whether smoking behaviors change after the onset of a chronic condition. We assessed the racial/ethnic differences in smoking behavior change after onset of chronic diseases among middle-aged and older adults in the US.

**Methods:**

We use longitudinal data from the Health and Retirement Study (HRS 1992–2010) to examine changes in smoking status and quantity of cigarettes smoked after a new heart disease, diabetes, cancer, stroke, or lung disease diagnosis among smokers.

**Results:**

The percentage of middle-aged and older smokers who quit after a new diagnosis varied by racial/ethnic group and disease: for white smokers, the percentage ranged from 14% after diabetes diagnosis to 32% after cancer diagnosis; for black smokers, the percentage ranged from 15% after lung disease diagnosis to 40% after heart disease diagnosis; the percentage of Latino smokers who quit was only statistically significant after stoke, where 38% quit. In logistic models, black (OR = 0.43, 95% CI: 0.19–0.99) and Latino (OR = 0.26, 95% CI: 0.11–0.65) older adults were less likely to continue smoking relative to white older adults after a stroke, and Latinos were more likely to continue smoking relative to black older adults after heart disease onset (OR = 2.69, 95% CI [1.05–6.95]). In models evaluating changes in the number of cigarettes smoked after a new diagnosis, black older adults smoked significantly fewer cigarettes than whites after a new diagnosis of diabetes, heart disease, stroke or cancer, and Latino older adults smoked significantly fewer cigarettes compared to white older adults after newly diagnosed diabetes and heart disease. Relative to black older adults, Latinos smoked significantly fewer cigarettes after newly diagnosed diabetes.

**Conclusions:**

A large majority of middle-aged and older smokers continued to smoke after diagnosis with a major chronic disease. Black participants demonstrated the largest reductions in smoking behavior. These findings have important implications for tailoring secondary prevention efforts for older adults.

**Electronic supplementary material:**

The online version of this article (doi:10.1186/s12877-017-0438-z) contains supplementary material, which is available to authorized users.

## Background

Higher mortality and morbidity burdens continue to disproportionately accrue to individuals from underrepresented racial and ethnic backgrounds into middle and old age [1–4]. Chronic disease complications are largely preventable with timely and effective care, and by having responsive disease management plans in place. Smoking is widely recognized as the main modifiable risk factor to prevent exacerbations of existing chronic disease and avert the development of incident chronic conditions. As a result, effecting changes in smoking behavior are among the first line of recommended actions to avert onset of cardiovascular disease, pulmonary disease, and various forms of cancer [5].

There are important differences in chronic disease morbidity and mortality for underrepresented racial and ethnic groups relative to whites. Both US-born Latinos and black Americans have greater smoking-related mortality than white Americans [5], and contend with significantly higher rates of cardiovascular and metabolic disease than whites even into older age [6]. Efforts to encourage behavior change after health problems have already set in (i.e., secondary prevention efforts) are critical to meeting public health goals of effective chronic disease management and health maintenance for older adult populations [7]. In addition, promotion of smoking cessation programs in late life may help in averting adverse events such as cancer recurrence, repeated heart attacks, and exacerbations in disease severity for older adult smokers that remain resistant to quitting [8–10]. A new chronic disease diagnosis could potentially serve as a warning, precipitating health behavior change and increased self-health vigilance [11, 12], or a “teachable moment” to intervene on adults with newly diagnosed chronic conditions [13–15]. Indeed, a small number of studies find that new health problems may actually improve the health and health habits of older adults [7, 10, 16].

There is little information about whether cessation attempts at older ages are more or less likely across racial and ethnic groups, and these differences may be an important source of disparities in health, including disease prevalence, prognosis after diagnosis, and quality of life.

While health behaviors are widely recognized as influential contributors to morbidity and mortality for the adolescent and young adult population [8], less attention has been focused on the effectiveness of smoking behavior modification among older adults [17–19]. Further, the adoption and maintenance of healthful behaviors—such as smoking reductions and cessation—may be of particular importance in the prevention of disease and compromised function associated with aging [18, 19].

The purpose of this study is to assess whether the onset of chronic disease elicits differential changes to smoking behaviors between black, Latino, and white older adults. To date, little is known about whether older adults from various racial and ethnic backgrounds are more or less likely to make lifestyle modifications in response to adverse changes to their health. These insights are critical in evaluating illness perception and behavior change as well as in assessing the adequacy and availability of secondary prevention efforts for underrepresented racial and ethnic older adults.

## Methods

### Data

This study uses data from the Health and Retirement Study (HRS), a nationally-representative sample of community-based adults over age 50 identified through screening of an area probability sample of households. The original HRS study cohort (born between 1942 and 1947) was first interviewed in 1992 and has since continued to be re-interviewed on a biennial basis. A detailed description of the HRS study design has been published elsewhere [20, 21] and publicly-available data files can be found on the HRS website (http://hrsonline.isr.umich.edu). The Institutional Review of the University of Michigan approved the Health and Retirement Study. As secondary data analysis of existing, de-identified survey data, the current study was exempt from human subject review by the Institutional Review Board of Oregon Health & Science University.

We examined data from 18,843 HRS respondents surveyed between the years of 1992–2010 who reported having at least one of the chronic conditions we studied (i.e., heart disease, diabetes, cancer, stroke, or lung disease) during the study period. 12,572 respondents reported a chronic condition that was not present at a previous wave. We made the following exclusions to arrive at our analytic sample: (1) 1014 respondents were age-ineligible spouses or had a zero sampling weights; (2) 549 respondents were missing in the pre-diagnosis wave (i.e., the immediately preceding wave from when the condition was reported); (3) 717 respondents reported their race as other/missing; and (4) 1243 respondents had missing smoking data. The resulting analytic sample consisted of 9049 individuals who were diagnosed with at least one of the chronic conditions of diabetes, heart disease, stroke, cancer, or lung disease at a follow-up interview.

### Design

This study included twelve waves of HRS data spanning 18 years (1992 to 2010). Analyses were based on all available waves of data for which items were worded consistently. We defined pre-illness diagnosis and post-illness diagnosis time points for each individual study respondent. The pre-diagnosis time point was defined as the last wave of interview prior to diagnosis. The post-diagnosis time point was defined as the same wave at which a respondent reported a new chronic disease, and, thus occurred between 0 and 2 years after the diagnosis. We included only participants who report a new diagnosis, and have valid smoking status (i.e., non-missing) in pre- and post diagnosis time points.

### Measures

#### Main outcome: current smoking status

The main outcome measure in this study was current smoking behavior, which was operationalized in two ways: (1) a binary indicator for current smoking status asked in the HRS questionnaire, “Do you smoke cigarettes now?” (1 = yes; 0 = no), and (2) a continuous variable for the number cigarettes smoked asked in the HRS questionnaire, “About how many cigarettes or packs do you usually smoke in a day now?” Smoking variables are available for all years of the survey to date (1992–2010).

#### Chronic disease indicators

We assessed changes in smoking behavior following new, incident disease. In order to ascertain disease status, the HRS asked study participants a series of questions beginning with, “Has a doctor ever told you that you had…” for the following list of conditions, “a heart attack, coronary heart disease, angina, congestive heart failure, or other heart problems?”; “diabetes or high blood sugar?”; “cancer or a malignant tumor of any kind except skin cancer?”; “stroke?”; and “chronic lung disease such as chronic bronchitis or emphysema?” Participants who indicated “yes” to any of these questions were considered to have a disease diagnosis for the corresponding disease. Each disease was examined separately regardless of the number of comorbid conditions present in participants with more than one disease diagnosis. Study participants with a new disease diagnosis following no disease diagnosis in the previous study wave were classified as having new disease onset.

#### Demographic and socioeconomic covariates

We used a set of dummy variables to represent differences among mutually-exclusive categories for non-Latino white (white), non-Latino black (black) and Latino individuals. We constructed dummy variables for white, black, and Latino included in the models to enable comparison between black vs. white, Latino vs. white, and Latino vs. black.

In addition to race and ethnicity, several demographic, socioeconomic, and health status factors were adjusted for in the analyses. We included age (number of years), female gender, education (high school graduate or not), income (in thousands of dollars), insurance coverage (insured or not), and marital status (partnered or not). Self-rated health was measured using a single question with five response categories prompting respondents to rate their own health as “excellent,” “very good,” “good,” “fair,” or “poor” (1–5, with 5 indicating “excellent” self-assessed health). Functional limitations were measured by difficulties with activities of daily living (e.g., dressing, bathing, eating) and instrumental activities of daily living (e.g., walking across a room, preparing a hot meal) assessed at the interview following a new diagnosis (“Because of a health problem do you have any difficulty…?”). Participants who indicated “yes” to any of the questions were considered to have some functional impairment. Because disease severity is not captured in the public release HRS data, including functional limitations and self-rated health as covariates may provide an indication of the severity of comorbid conditions that study participants contend with. Comorbidity was included as a total count of additional chronic conditions (heart disease, diabetes, stroke, cancer, lung disease, hypertension, and arthritis) for individuals prior to being diagnosed with the new condition in question. Age, gender, education, income, and marital status were measured at the pre-diagnosis time point. Income, insurance coverage, functional limitations, self-rated health, and comorbidity were measured at the post-diagnosis time point.

### Analysis

All statistical analyses were weighted for nonresponse and post-stratification and adjusted for complex sampling with SAS software, version 9.2 (SAS Institute Inc., Carey, North Carolina) according to recommendations for analysis of the HRS [21]. Because standard procedures used to test for repeated measures differences in proportions, such as the McNemar’s chi-square, are not available with software used for sampling design adjustments, initial tests of differences in smoking proportions without covariates were made using the Rao-Scott chi-square [22] comparing discordant cells (i.e., 0-1 vs. 1-0 responses) [23]. Paired t-tests were used to compare pre- and post-diagnosis means for continuous variables [24].

Logistic models were estimated to predict racial/ethnic differences in smoking status change after the onset of a chronic condition among former smokers and nonsmokers. Additional analyses examined the number of cigarettes smoked per day only among smokers. We estimated negative binomial models to account for observed overdispersion in the distribution of the number of cigarettes smoked [25] and report the exponentiated coefficients (ratio of means, or incident rate ratios). These models investigated racial/ethnic differences in the quantity of cigarettes smoked after chronic disease diagnosis among smokers (i.e., respondents that reported consistently smoking in all previous periods of observation). In both sets of models (logistic and negative binomial), we estimate smoking behaviors in the post-diagnosis period controlling for pre-diagnosis smoking and differences in gender, age, education, functional limitations, self-rated health, and comorbidity burden by race/ethnic group membership.

## Results

We identified 9049 middle-aged and older adults that were included in the analysis (Table 1). In our study, 76% were white, 15% black, and 9% Latino. A slight minority of respondents were female (48.9%) and most had at least high school education level (75.2%). At the first post-diagnosis wave, respondents had an average age was 67.2 years, with a mean of 4.7 functional limitations, and 1.7 co-occurring chronic diseases.

**Table 1:** Descriptive characteristics, HRS analytic sample 1992–2010

Measures	Total *N* = 9049	White *n* = 6903 (76%)	Black *n* = 1368 (15%)	Latino *n* = 778 (9%)
New Diagnoses				
Heart Disease, %	44.3	45.1	39.6	40.6
Lung Disease, %	22.2	22.8	21.0	15.1
Cancer, %	27.7	28.7	26.0	16.9
Diabetes, %	31.0	28.9	38.5	46.6
Stroke, %	14.7	14.2	18.5	15.0
Age at diagnosis, mean	67.2	67.7	64.9	65.0
Female, %	48.9	48.1	54.5	49.8
High school graduate, mean	75.2	80.4	57.2	35.3
Income (thousands of dollars), mean	52.7	56.9	34.4	26.1
Insured, %	94.7	96.0	91.3	82.0
ADL/IADL score (0–11), mean	4.7	4.6	5.6	5.3
Comorbidity (0–7), mean	1.7	1.7	2.0	1.7
SRH (1–5)				
Poor, %	14.8	13.7	20.6	21.0
Fair, %	28.4	26.1	37.8	43.8
Good, %	33.6	34.9	27.4	26.0
Very Good, %	19.1	20.8	12.0	7.4
Excellent, %	4.1	4.5	2.3	1.8

Covariate measures are assessed at the post-diagnosis time period. Means and proportions are weighted (post-diagnosis weight) and adjusted for complex sampling design. Comorbidity is a count of self-reported arthritis, hypertension, diabetes, stroke, heart disease, lung disease and cancer

Acronyms: *HRS* Health and Retirement Study, *ADL* activities of daily living, *IADL* instrumental activities of daily living, *SRH* self-rated health

### Within-race/ethnic group smoking changes after diagnosis

Table 2 presents weighted percentages, weighted counts at pre-diagnosis and post-diagnosis (between 0 and 2 years following diagnosis) and percent change (i.e., percent who quit for the current smoking outcome, and changes in the mean number of cigarettes smoked) for white, black, and Latino respondents. These pre- and post-diagnosis measures reflect the within race/ethnic group temporal changes in smoking. Overall, reductions in current smoker percentages were evident across all three racial/ethnic groups after chronic disease onset, with the exception of Latino respondents after diabetes onset. The percentage of older adults who quit smoking after a new diagnosis varied widely by racial/ethnic group and disease. The percentage of white smokers who quit ranged from 14% after diabetes diagnosis to 32% after cancer diagnosis. For black smokers, the percentage who quit ranged from 15% after lung disease diagnosis to 40% after heart disease diagnosis. The percentage of Latino smokers who quit was only statistically significant after stoke, where 38% quit.

**Table 2:** Within racial/ethnic group change in smoking in the period preceding and following chronic disease diagnosis, HRS 1992–2010

	White				Black				Latino			
	N	Pre	Post	% Change	N	Pre	Post	% Change	N	Pre	Post	% Change
Diabetes												
Current Smoker (%)	1954	19.7	16.9**	−14.2	526	27.1	22.0**	−18.7	354	15.5	15.6	+1.0
# Cigarettes (mean)	351	18.3	15.7*	−14.0	126	12.8	10.2	−20.2	54	8.4	7.8	−6.2
Heart disease												
Current Smoker (%)	3142	20.7	14.3**	−30.9	536	30.2	18.1**	−40.1	312	22.1	18.8	−15.0
# Cigarettes (mean)	607	18.4	11.5**	−37.5	140	12.1	6.0**	−50.5	55	9.8	9.5	−3.6
Stroke												
Current Smoker (%)	1024	22.5	18.6**	−17.5	264	32.9	23.3**	−29.2	128	30.4	19.0**	−37.6
# Cigarettes (mean)	202	18.1	14.9**	−17.8	69	13.3	9.4*	−29.3	33	11.5	10.1	−12.2
Cancer												
Current Smoker (%)	1963	20.7	14.1**	−32.0	357	30.0	21.4**	−28.6	154	22.1	15.2	−31.2
# Cigarettes (mean)	278	18.9	12.0**	−36.5	55	11.9	7.8**	−34.2	20	11.7	9.8**	−16.1
Lung disease												
Current Smoker (%)	1539	41.7	33.8**	−18.9	273	45.8	38.8**	−15.3	119	36.6	34.8	−5.0
# Cigarettes (mean)	613	19.4	14.0**	−27.9	111	11.1	11.3	+1.7	36	13.7	7.9**	−42.4

Within-group change in health behavior was assessed using Rao–Scott chi square tests to compare pre- and post-diagnosis proportions for categorical outcomes and paired t-tests to compare pre- and post-diagnosis means for continuous outcomes. All analyses are weighted (post-diagnosis weight) and adjusted for complex sampling design using SAS 9.2. The “% Change” column represents percentage who quit for the current smoker outcome and changes in cigarettes smoked for # cigarettes outcome. Statistical test significance levels: * < .05; ** < .01

Acronym: *HRS* Health and Retirement Study

Among respondents who continued to smoke after a chronic disease diagnosis, significant reductions in the mean number of cigarettes smoked were observed for white middle-aged and older adults across any of the five chronic diseases. Black respondents significantly reduced mean cigarette consumption after newly diagnosed heart disease, stroke, and cancer. Latino respondents significantly reduced the quantity of cigarettes smoked after cancer and lung disease.

### Between-race/ethnic group smoking changes after diagnosis

Table 3 presents the estimated changes in current smoking status after disease diagnosis controlling for differences in demographic characteristics, socioeconomic factors, and health status covariates for black and Latino relative to white respondents. We also estimate the same model in Additional file 1: Table A1, with black as the race/ethnic reference group. Pre-diagnosis smoking was included to account for initial racial/ethnic differences in smoking. The logistic regression provides the odds of smoking post-diagnosis given the odds of smoking initially after controlling for covariates. Relative to middle-aged and older whites, black (OR = 0.43, 95% CI: 0.19–0.99) and Latino (OR = 0.26, 95% CI: 0.11–0.65) older adults were less likely to continue smoking after a new onset of stroke. From Additional file 1: Table A1, relative to black middle-aged and older adults, Latinos were more likely to continue smoking after newly diagnosed heart disease (OR = 2.69, 95% CI: 1.05–6.95).

**Table 3:** Between racial/ethnic group^a^ change in smoking in the period preceding and following chronic disease diagnosis, HRS 1992–2010

	Diabetes		Heart Disease		Stroke		Cancer		Lung Disease	
	Post- diagnosis smoker (*n* = 2796)		Post- diagnosis smoker (*n* = 4055)		Post- diagnosis smoker (*n* = 1515)		Post- diagnosis smoker (*n* = 2434)		Post- diagnosis smoker (*n* = 1978)	
	OR	95% CI	OR	95% CI	OR	95% CI	OR	95% CI	OR	95% CI
White (reference group)	-	-	-	-	**-**	-	-	-	-	-
Black	0.75	0.44–1.32	0.69	0.41–1.15	0.43*	**0.19–0.99**	0.9	0.45–1.80	0.98	0.52–1.84
Latino	1.02	0.52–1.98	1.86	0.77–4.52	0.26**	**0.11–0.65**	0.54	0.18–1.58	1.09	0.41–2.89
Age (years)	0.97*	0.94–0.99	0.97**	0.95–0.99	0.95**	0.92–0.98	0.98	0.95–1.00	0.97**	0.95–0.99
Female	0.92	0.60–1.42	1.67**	1.20–2.34	1.00	0.59–1.69	1.00	0.65–1.52	1.44	0.99–2.09
Education (years)	0.98	0.91–1.06	1.01	0.95–1.00	0.89**	0.82–0.96	0.92	0.85–1.00	0.95	0.89–1.01
Income (thousands)	1.00	0.99–1.00	1.00	0.99–1.00	0.99	0.98–1.00	0.99*	0.98–0.99	1.00	0.99–1.00
Insured	1.17	0.38–3.60	1.09	0.52–2.31	1.22	0.41–3.61	0.59	0.22–1.58	0.68	0.34–1.35
ADL/IADL score (0–11)	1.01	0.92–1.10	0.95	0.89–1.01	0.95	0.86–1.04	0.90*	0.83–0.98	0.95	0.89–1.02
Comorbidity (0–7)	0.84	0.66–1.07	0.92	0.79–1.08	1.00	0.79–1.26	1.16	0.94–1.42	0.90	0.75–1.08
SRH (1–5)	0.79	0.61–1.02	0.92	0.75–1.14	0.9	0.65–1.24	0.90	0.73–1.12	0.92	0.74–1.15
Pre-diagnosis smoker	113.06**	69.25–184.60	109.18**	70.77–168–45	105.76**	55.01–203.37	82.53**	47.14–144.51	70.38**	45.74–108.31

All model covariates as assessed at the post-diagnosis time period. All analyses were weighted (post-diagnosis weight) and adjusted for complex sampling design using SAS 9.2. Statistically significant racial/ethnic differences in smoking change are bolded. Binary models estimate post-diagnosis smoking behavior controlling for pre-diagnosis status

Acronyms: *HRS* Health and Retirement Study, *OR* odds ratios, *IRR* incidence rate ratios

^a^White as reference group. Statistical test significance levels: * < .05; ** < .01

Table 4 presents the changes in the number of cigarettes smoked post-diagnosis for black and Latino relative to white smokers after controlling for the number of cigarettes smoked in the pre-period as well as socio-demographic and health status covariates. We also estimate the same model in Additional file 2: Table A2, with black as the race/ethnic reference group. Relative to white middle-aged and older adults, black respondents smoked 0.7 times fewer (95% CI: 0.54–0.91) cigarettes after a new diagnosis of diabetes, 0.54 times fewer (95% CI: 0.41–0.72) cigarettes after new heart disease, 0.53 times fewer (95% CI: 0.35–0.80) cigarettes after new stroke, and 0.67 times fewer (95% CI: 0.47–0.96) cigarettes after new cancer onset. Relative to white middle-aged and older adults, Latino respondents smoked 0.42 times fewer (95% CI: 0.29–0.62) cigarettes after newly diagnosed diabetes and 0.63 times fewer (95% CI: 0.44–0.90) cigarettes after heart disease onset. From Additional file 2: Table A2, relative to black middle-aged and older adults, Latino respondents smoked 0.6 times fewer (95% CI: 0.41–0.89) cigarettes after newly diagnosed diabetes.

**Table 4:** Between racial/ethnic group^a^ change in number of cigarettes smoked among smokers in the period preceding and following chronic disease diagnosis, HRS 1992–2010

	Diabetes		Heart Disease		Stroke		Cancer		Lung Disease	
	Post-diagnosis cigarettes smoked (*n* = 490)		Post-diagnosis cigarettes smoked (*n* = 832)		Post-diagnosis cigarettes smoked (*n* = 317)		Post-diagnosis cigarettes smoked (*n* = 348)		Post-diagnosis cigarettes smoked (*n* = 758)	
	IRR	95% CI	IRR	95% CI	IRR	95% CI	IRR	95% CI	IRR	95% CI
White (reference group)	**-**	-	**-**	-	**-**	-	**-**	-	-	-
Black	0.70**	**0.54–0.91**	0.54**	**0.41–0.72**	0.53**	**0.35–0.80**	0.67*	**0.47–0.96**	1.02	0.77–1.35
Latino	0.42**	**0.29–0.62**	0.63*	**0.44–0.90**	0.78	0.40–1.53	0.89	0.47–1.68	0.62	0.35–1.10
Age (years)	0.98*	0.97–0.99	0.98*	0.97–0.99	1.00	0.98–1.02	0.98	0.96–1.00	0.99	0.98–1.00
Female	0.98	0.79–1.21	1.14	0.93–1.39	0.9	0.69–1.17	0.79	0.59–1.05	0.95	0.80–1.11
Education (years)	0.99	0.96–1.03	0.97*	0.94–0.99	1.00	0.96–1.05	0.95	0.91–1.00	0.98	0.95–1.02
Income (thousands)	1.00	0.99–1.00	1.00	0.99–1.00	1.00	0.99–1.00	0.99*	0.99–0.99	1.00	0.99–1.00
Insured	1.08	0.65–1.79	1.16	0.81–1.68	1.31	0.80–2.15	1.05	0.73–1.50	0.99	0.81–1.20
ADL/IADL score (0–11)	0.98	0.95–1.02	1.01	0.97–1.05	1.07*	1.01–1.13	0.98	0.93–1.03	0.96*	0.92–0.99
Comorbidity (0–7)	0.92	0.83–1.03	1.00	0.91–1.09	1.04	0.93–1.15	1.00	0.89–1.13	1.04	0.96–1.13
SRH (1–5)	0.87*	0.77–0.99	1.00	0.89–1.12	1.12	0.92–1.36	1.03	0.84–1.14	0.98	0.88–1.10
Pre-diagnosis cigarettes smoked	1.02**	1.01–1.03	1.02**	1.01–1.03	1.03**	1.02–1.04	1.02**	1.02–1.04	1.03**	1.02–1.04

All model covariates as assessed at the post-diagnosis time period. All analyses were weighted (post-diagnosis weight) and adjusted for complex sampling design using SAS 9.2. Statistically significant racial/ethnic differences in smoking change are bolded

Acronyms: *HRS* Health and Retirement Study, *OR* odds ratios, *IRR* incidence rate ratios

^a^White as reference group. Statistical test significance levels: * < .05; ** < .01

## Discussion

The objective of this study was to investigate changes in smoking behavior between black, Latino, and white adults after a chronic illness diagnosis. Moderate-to-low percentages of middle-aged and older adults quit smoking after the onset of a new chronic disease diagnosis, and these varied widely by race/ethnic group and by chronic disease type. Similarly, we found modest reductions in the quantity of cigarettes smoked for middle-aged and older adults who continued to smoke after a newly diagnosed chronic disease, and this too varied widely by race/ethnic group and chronic disease type. Although observed reductions represent some positive change to smoking behavior among older adults, the majority of respondents continued to smoke after being diagnosis with a new illness.

The smoking patterns of older adults have been largely ignored by researchers, who have focused predominantly on younger adult smoking behaviors [26]. Among non-elderly adults, whites are more likely to quit at any given time point, and importantly, black Americans—who predominantly smoke mentholated cigarettes—have lower cessation rates and experience higher rates of smoking-related health consequences relative to white Americans despite smoking fewer cigarettes, on average [27–29]. This literature provides a useful counterpoint to our findings for middle-aged and older Americans of relatively low smoking cessation across race/ethnic groups in the face of health declines.

We found several interesting differences in smoking behavior change between black, Latino, and white older adults. Relative to whites, black and Latino respondents were less likely to smoke after suffering a stroke. Stated conversely, older white adults were less likely to quit after stroke than were black and Latino older adults. Comparing changes in smoking behavior between black and Latino middle-aged and older adults, Latinos were less likely to quit smoking after newly diagnosed heart disease. Further, white middle-aged and older adults smoked the greatest quantity of cigarettes despite health downturns. Relative to whites, black participants smoked fewer cigarettes after a new diabetes, heart disease, stroke, or cancer diagnosis, and Latinos smoked fewer cigarettes after a new diagnosis of diabetes or heart disease. These differences may have important health consequences for older adults—particularly older white adults—who continue to smoke after chronic disease onset, which could lead to exacerbations and complications of the disease, or other serious adverse events.

Efforts to promote health behavior change for individuals from socioeconomically and ethnically diverse backgrounds have generated a nuanced discussion in the literature. On one hand, high-risk and underserved populations should be a focus of prevention efforts given lower life expectancy in the US, greater likelihood of cancer mortality, and greater likelihood of engaging in risky health behaviors [30]. In addition, high prevalence of risky health behaviors—smoking in particular—among disadvantaged older adults may contribute to maintaining inequalities in mortality and morbidity into middle and old age [19]. Our study explicitly compared changes in smoking behavior between white, black, and Latino older adults, and we did not find that blacks or Latinos were less likely to quit or reduce smoking levels relative to whites. Our findings provide support for better access to smoking cessation counseling and therapies for middle-aged and older adults regardless of race/ethnic background, but may warrant particular emphasis on whites whom we found less likely to quit smoking after suffering a stroke, and who smoke 1.5 to nearly 2 times as many cigarettes as blacks after newly diagnosed diabetes, cancer, heart disease or stroke.

Our findings suggest there may be differences in smoking behavior change after the diagnosis of specific chronic diseases, but we could not ascertain why or how this occurred. Based on other research, we can speculate about several potential explanations. There may be differences in the importance assigned to the diffusion of smoking cessation counseling by clinicians in the face of critical health events, such as a stroke. In addition, the ability to react to adverse health events and assimilate health warnings brought on by a recent diagnosis or health decline may be constrained for some individuals. Lack of access to timely smoking cessation counseling may have important repercussions for secondary prevention efforts to avert poor health outcomes for older adults [31]. While our results do not speak to the causes of continuing, stopping, or reducing smoking behaviors, our overall findings imply that middle-aged and older adults may have different patterns of smoking, may have other deterrents to stopping smoking, may not be effectively targeted by smoking interventions, or may have different perspectives on the effects of smoking on health [31].

This study contributes to the understanding of health behaviors in important ways, and draws from a number of strengths. First, the prospective study design assesses health behaviors prior to the diagnosis of the condition and is less susceptible to recall and social desirability biases. Second, this study compares lifestyle changes among the most serious chronic conditions, an improvement over many earlier studies that examine health conditions in isolation. Third, the consideration of racial and ethnic group differences in smoking behavior change in response to chronic disease onset provides important insights on reducing subsequent health risks for white, black, and Latino older adults. Finally, this study contributes to the understanding of whether behavioral processes are similar or different for minority and white individuals and where improvements in chronic disease management are most needed.

Several limitations should also be noted. First, our measures of health conditions, including disease diagnosis and smoking behaviors were derived from self- report. To the extent that there is under-reporting of health conditions [32], any bias would likely overestimate changes in smoking behavior, because individuals with pre-clinical indications of disease (e.g., pre-diabetes) would not have been included and might be less likely to receive repeated smoking cessation messaging or would be less motivated to change. However, several studies have shown reasonable concordance between self-reported chronic conditions and other methods of ascertainment [33–35]. Second, the present study is not a matched-cohort controlled study, therefore we should be cautious in attributing changes in smoking behavior to new disease onset. However, the objective of our study was not to examine smoking cessation outcomes relative to controls who are not diagnosed with chronic disease. There is a higher likelihood of cessation that has been shown relative to controls in a previous study [18]. In the present study, we instead compare smoking changes over a 2-year window around a new chronic disease diagnosis between black, white, and Latino middle-aged and older adults in the US. Third, our data do not permit examination of quit attempts in our study. Cessation attempts may vary for individuals from underrepresented racial/ethnic background due to compounding influences with poor socioeconomic status, low literacy levels, lack of access to environments that are conducive to healthful lifestyles, not receiving important health messages from their health providers that are delivered in appropriate ways, and lack of access to and use of smoking cessation therapies. Further, success in quitting (i.e., lack of re-initiation) is more likely to occur for adults with greater socioeconomic resources [36]. While we cannot assess cessation attempts, our study examines cessation and changes in smoking amounts around a critical clinical diagnostic window, which represents useful and actionable information to intervene on older smokers after they have developed a chronic condition. Finally, our study involves new diagnosis of serious health conditions; however, it is unknown whether individuals made lifestyle changes prior to diagnosis. Some individuals may reduce or quit smoking after receiving health warnings such as repeated high blood pressure or cholesterol measurements. Inclusion of medical record information of pre-diagnosis risk factors along with subsequent diagnosis of major conditions in future studies would provide important new information about whether or when individuals change behavior at earlier points of disease development.

There may be important extensions to this study that should be addressed in future work. While our study examined differences in changes to smoking after chronic disease onset between white, black, and Latino Americans, it is also important to understand the predictors and determinants of changes to smoking behaviors for each of these racial and ethnic background groups. Future studies should address important factors that predict cessation and smoking reductions for black, white, and Latino older adults. In addition, there may be important dynamics to smoking cessation and smoking reductions for middle-aged and older men and women, and for men and women from different racial/ethnic backgrounds. These considerations should also be addressed in future studies.

## Conclusions

Overall, the post-diagnosis reductions in smoking for white and black adults in middle and late life are encouraging, and indicate that the onset of a diagnosis may be an actionable or “teachable moment” for smokers to change. Onset of a new chronic condition is not an uncommon occurrence for middle-aged and older adults and should provide an important opportunity to address smoking cessation in the clinical setting at the time of a new diagnosis. Greater efforts should be made to reach older adults once chronic diseases have set in, possibly through increased efforts at reaching these populations with access to appropriate smoking cessation messaging and therapies. Additional research can identify the most effective strategies for cessation interventions in the context of incident chronic disease.

## Additional files

Additional file 1: Table A1. Between racial/ethnic group^a^ change in smoking in the period preceding and following chronic disease diagnosis, HRS 1992-2010. (DOCX 40 kb)
Additional file 2: Table A2. Between racial/ethnic group^a^ change in number of cigarettes smoked among smokers in the period preceding and following chronic disease diagnosis, HRS 1992-2010. (DOCX 34 kb)

## Abbreviations

ADL: Activities of daily living
HRS: Health and Retirement Study
IADL: Instrumental activities of daily living
IRR: Incidence rate ratio
OR: Odds ratio
SRH: Self-rated health
