# Supplementary material for: Racial and ethnic differences in smoking changes after chronic disease diagnosis among middle-aged and older adults in the United States
Source: BMC Geriatr. 2017 Feb 8;17:48. doi: 10.1186/s12877-017-0438-z (PMC5299693; doi:10.1186/s12877-017-0438-z)
Supplement: Additional file 1: Table A1. — Between racial/ethnic groupa change in smoking in the period preceding and following chronic disease diagnosis, HRS 1992-2010. (DOCX 40 kb) [file 12877_2017_438_MOESM1_ESM.docx]

| Additional file 1: Table A1. Between racial/ethnic group^†^ change in smoking in the period preceding and following chronic disease diagnosis, HRS 1992-2010 | | | | | | | | | | |
| --- | --- | --- | --- | --- | --- | --- | --- | --- | --- | --- |
|  | **Diabetes** | | **Heart Disease** | | **Stroke** | | **Cancer** | | **Lung Disease** | |
|  | Post- diagnosis smoker  (n=2796) | | Post- diagnosis smoker  (n=4055) | | Post- diagnosis smoker  (n=1515) | | Post- diagnosis smoker  (n=2434) | | Post- diagnosis smoker  (n=1978) | |
|  | **OR** | **95% CI** | **OR** | **95% CI** | **OR** | **95% CI** | **OR** | **95% CI** | **OR** | **95% CI** |
| Black (reference group) | - | - | - | - | **-** | - | - | - | - | - |
| White | 1.33 | 0.76-2.30 | 1.45 | 0.86-2.42 | **2.31*** | **1.01-5.31** | 1.10 | 0.56-2.21 | 1.02 | 0.55-1.91 |
| Latino | 1.35 | 0.61-2.96 | **2.69*** | **1.05-6.95** | 0.61 | 0.21-1.81 | 0.59 | 0.17-1.99 | 1.11 | 0.37-3.36 |
| Age (years) | 0.97* | 0.94-0.99 | 0.97** | 0.95-0.99 | 0.95** | 0.92-0.98 | 0.98 | 0.95-1.00 | 0.97** | 0.95-0.99 |
| Female | 0.92 | 0.60-1.42 | 1.67** | 1.20-2.34 | 1.00 | 0.59-1.69 | 1.00 | 0.65-1.52 | 1.44 | 0.99-2.09 |
| Education (years) | 0.98 | 0.91-1.06 | 1.01 | 0.95-1.00 | 0.89** | 0.82-0.96 | 0.92 | 0.85-1.00 | 0.95 | 0.89-1.01 |
| Income (thousands) | 1.00 | 0.99-1.00 | 1.00 | 0.99-1.00 | 0.99 | 0.98-1.00 | 0.99* | 0.98-0.99 | 1.00 | 0.99-1.00 |
| Insured | 1.17 | 0.38-3.60 | 1.09 | 0.52-2.31 | 1.22 | 0.41-3.61 | 0.59 | 0.22-1.58 | 0.68 | 0.34-1.35 |
| ADL/IADL score (0-11) | 1.01 | 0.92-1.10 | 0.95 | 0.89-1.01 | 0.95 | 0.86-1.04 | 0.90* | 0.83-0.98 | 0.95 | 0.89-1.02 |
| Comorbidity (0-7) | 0.84 | 0.66-1.07 | 0.92 | 0.79-1.08 | 1.00 | 0.79-1.26 | 1.16 | 0.94-1.42 | 0.90 | 0.75-1.08 |
| SRH (1-5) | 0.79 | 0.61-1.02 | 0.92 | 0.75-1.14 | 0.9 | 0.65-1.24 | 0.90 | 0.73-1.12 | 0.92 | 0.74-1.15 |
| Pre-diagnosis smoker | 113.06** | 69.25-184.60 | 109.18** | 70.77-168-45 | 105.76** | 55.01-203.37 | 82.53** | 47.14-144.51 | 70.38** | 45.74-108.31 |
| Acronyms: HRS=Health and Retirement Study; OR=odds ratios; IRR=incidence rate ratios.  Notes: All model covariates as assessed at the post-diagnosis time period. All analyses were weighted (post-diagnosis weight) and adjusted for complex sampling design using SAS 9.2. Statistically significant racial/ethnic differences in smoking change are bolded. Binary models estimate post-diagnosis smoking behavior controlling for pre-diagnosis status. ^†^Black as reference group. Statistical test significance levels: * <.05; **<.01 | | | | | | | | | | |
|  |  |  |  |  |  |  |  |  |  |  |

| Supplemental Table A2. Between racial/ethnic group^†^ change in number of cigarettes smoked among smokers in the period preceding and following  chronic disease diagnosis, HRS 1992-2010 | | | | | | | | | | |
| --- | --- | --- | --- | --- | --- | --- | --- | --- | --- | --- |
|  | **Diabetes** | | **Heart Disease** | | **Stroke** | | **Cancer** | | **Lung Disease** | |
|  | Post-diagnosis cigarettes smoked  (n=490) | | Post-diagnosis  cigarettes smoked  (n=832) | | Post-diagnosis  cigarettes smoked  (n=317) | | Post-diagnosis cigarettes smoked  (n=348) | | Post-diagnosis cigarettes smoked  (n=758) | |
|  | **IRR** | **95% CI** | **IRR** | **95% CI** | **IRR** | **95% CI** | **IRR** | **95% CI** | **IRR** | **95% CI** |
| Black (reference group) | **-** | - | **-** | - | **-** | - | **-** | - | - | - |
| White | **1.45**** | 1.10-1.85 | **1.85**** | 1.39-2.45 | **1.89**** | 1.26-2.83 | **1.49*** | 1.04-2.14 | 0.99 | 0.74-1.30 |
| Latino | **0.60*** | 0.41-0.89 | 1.16 | 0.78-1.72 | 1.47 | 0.75-2.88 | 1.32 | 0.67-2.63 | 0.61 | 0.34-1.12 |
| Age (years) | 0.98* | 0.97-0.99 | 0.98* | 0.97-0.99 | 1.00 | 0.98-1.02 | 0.98 | 0.96-1.00 | 0.99 | 0.98-1.00 |
| Female | 0.98 | 0.79-1.21 | 1.14 | 0.93-1.39 | 0.9 | 0.69-1.17 | 0.79 | 0.59-1.05 | 0.95 | 0.80-1.11 |
| Education (years) | 0.99 | 0.96-1.03 | 0.97* | 0.94-0.99 | 1.00 | 0.96-1.05 | 0.95 | 0.91-1.00 | 0.98 | 0.95-1.02 |
| Income (thousands) | 1.00 | 0.99-1.00 | 1.00 | 0.99-1.00 | 1.00 | 0.99-1.00 | 0.99* | 0.99-0.99 | 1.00 | 0.99-1.00 |
| Insured | 1.08 | 0.65-1.79 | 1.16 | 0.81-1.68 | 1.31 | 0.80-2.15 | 1.05 | 0.73-1.50 | 0.99 | 0.81-1.20 |
| ADL/IADL score (0-11) | 0.98 | 0.95-1.02 | 1.01 | 0.97-1.05 | 1.07* | 1.01-1.13 | 0.98 | 0.93-1.03 | 0.96* | 0.92-0.99 |
| Comorbidity (0-7) | 0.92 | 0.83-1.03 | 1.00 | 0.91-1.09 | 1.04 | 0.93-1.15 | 1.00 | 0.89-1.13 | 1.04 | 0.96-1.13 |
| SRH (1-5) | 0.87* | 0.77-0.99 | 1.00 | 0.89-1.12 | 1.12 | 0.92-1.36 | 1.03 | 0.84-1.14 | 0.98 | 0.88-1.10 |
| Pre-diagnosis cigarettes smoked | 1.02** | 1.01-1.03 | 1.02** | 1.01-1.03 | 1.03** | 1.02-1.04 | 1.02** | 1.02-1.04 | 1.03** | 1.02-1.04 |
| Acronyms: HRS=Health and Retirement Study; OR=odds ratios, IRR=incidence rate ratios.  Notes: All model covariates as assessed at the post-diagnosis time period. All analyses were weighted (post-diagnosis weight) and adjusted for complex sampling design using SAS 9.2. Statistically significant racial/ethnic differences in smoking change are bolded. ^†^Black as reference group. Statistical test significance levels: * <.05; **<.01 | | | | | | | | | | |
|  |  |  |  |  |  |  |  |  |  |  |
